# Quantitative motor assessment of muscular weakness in myasthenia gravis: a pilot study

**DOI:** 10.1186/s12883-015-0517-8

**Published:** 2015-12-23

**Authors:** Sarah Hoffmann, Jana Siedler, Alexander U. Brandt, Sophie K. Piper, Siegfried Kohler, Christian Sass, Friedemann Paul, Ralf Reilmann, Andreas Meisel

**Affiliations:** NeuroCure Clinical Research Center, Charité - Universitätsmedizin Berlin, Charitéplatz 1, 10117 Berlin, Germany; Department of Neurology, Charité - Universitätsmedizin Berlin, Charitéplatz 1, 10117 Berlin, Germany; Centrum für Schlaganfallforschung Berlin (CSB), Klinik für Neurologie, Charité - Universitätsmedizin Berlin, Charitéplatz 1, 10117 Berlin, Germany; George-Huntington-Institute, Technology-Park, Johann-Krane Weg 27, 48149 Muenster, Germany; Department of Radiology, Universitätsklinikum Münster, Albert-Schweitzer Campus 1, 48149 Muenster, Germany; Department of Neurodegenerative Diseases and Hertie-Institute for Clinical Brain Research, University of Tuebingen, Hoppe-Seyler Str. 3, 72076 Tuebingen, Germany; Department of Neurology, Asklepios Klinikum Harburg, Eißendorfer Pferdeweg 52, 21075 Hamburg, Germany

**Keywords:** Myasthenia gravis, Neurophysiology, Case control studies, Diagnostic tests, Clinical trial

## Abstract

**Background:**

Muscular weakness in myasthenia gravis (MG) is commonly assessed using Quantitative Myasthenia Gravis Score (QMG). More objective and quantitative measures may complement the use of clinical scales and might detect subclinical affection of muscles. We hypothesized that muscular weakness in patients with MG can be quantified with the non-invasive Quantitative Motor (Q-Motor) test for Grip Force Assessment (QGFA) and Involuntary Movement Assessment (QIMA) and that pathological findings correlate with disease severity as measured by QMG.

**Methods:**

This was a cross-sectional pilot study investigating patients with confirmed diagnosis of MG. Data was compared to healthy controls (HC). Subjects were asked to lift a device (250 and 500 g) equipped with electromagnetic sensors that measured grip force (GF) and three-dimensional changes in position and orientation. These were used to calculate the position index (PI) and orientation index (OI) as measures for involuntary movements due to muscular weakness.

**Results:**

Overall, 40 MG patients and 23 HC were included. PI and OI were significantly higher in MG patients for both weights in the dominant and non-dominant hand. Subgroup analysis revealed that patients with clinically ocular myasthenia gravis (OMG) also showed significantly higher values for PI and OI in both hands and for both weights. Disease severity correlates with QIMA performance in the non-dominant hand.

**Conclusion:**

Q-Motor tests and particularly QIMA may be useful objective tools for measuring motor impairment in MG and seem to detect subclinical generalized motor signs in patients with OMG. Q-Motor parameters might serve as sensitive endpoints for clinical trials in MG.

## Background

Myasthenia gravis is an autoimmune mediated disease of the neuromuscular junction with fluctuating muscle weakness as cardinal symptom [1, 2]. The weakness can affect all voluntary (striated) muscle groups with great intra- and inter-individual variability. Assessment of muscle weakness is crucial for clinical monitoring of MG patients, evaluation of treatment success and as outcome parameter in clinical trials. The distribution and severity of muscle weakness is commonly assessed using the Myasthenia Gravis Foundation of America Clinical Classification (MGFA) [3] and the Quantitative Myasthenia Gravis Score (QMG) [4]. Currently, instrument-based diagnostics include electrophysiological tests, namely repetitive nerve stimulation (RNS) and single-fiber electromyography (SFEMG). However, both tests have their limitations with RNS being insufficiently sensitive in ocular myasthenia gravis (OMG) [5] and SFEMG often being unpleasant and painful for the patients and needing an experienced examiner [6].

We therefore hypothesized that muscular weakness in patients with myasthenia gravis can be objectively quantified with non-invasive quantitative motor (Q-Motor) grip force assessment (QGFA) and involuntary movement assessment (QIMA) and that pathological findings correlate with disease severity as measured by the QMG. Furthermore, we wanted to explore if patients with purely ocular symptoms show subclinical signs of generalized muscle weakness compared to healthy controls.

## Patients and methods

### Patients

This is a cross-sectional study that included patients with confirmed diagnosis of myasthenia gravis independent of disease duration and severity (excluding myasthenic crisis). Patients were consecutively screened in our outpatient clinic between March 2011 and May 2012. All data was compared to a group of healthy controls (HC) that was similar in age and sex (distribution) to the patient group. HC had to fulfil the following criteria: Age ≥18 years, no other neurological diseases, no other diseases affecting the musculoskeletal system, no cognitive deficits. Overall, 40 patients with MG and 23 HC were included.

### Clinical assessment

Patients were examined under supervision of a board certified neurologist. Sociodemographics as well as current medication were documented. The handedness of MG patients and HC were considered in all motor tasks and separately analyzed (dominant and non-dominant hand). For clinical assessment we used the MGFA classification and the QMG score. The classification of the Myasthenia Gravis Foundation of America (MGFA) is designed to identify subgroups of patients with MG who share distinct clinical features or severity of disease [3]. Using the MGFA classification, patients were grouped into ocular (MGFA I) or generalized MG patients (MGFA II-IV). Within the group of patients with generalized MG, we distinguished between patients with muscle weakness predominantly affecting limb and/ or axial muscles (MGFA II-IVa) and patients with muscle disease predominantly affecting oropharyngeal and/ or respiratory muscles (MGFA II-IVb). Disease severity was assessed using the QMG score. The QMG score was developed as a tool for assessing disease severity as well as the pattern of deficits based on quantitative testing of sentinel muscle groups [3, 7]. It is a 13-item score with a total score range of 0–39 points and shows good interrater variability [4].

### Quantitative motor grip force assessment and involuntary movement assessment

**Fig. 1:**
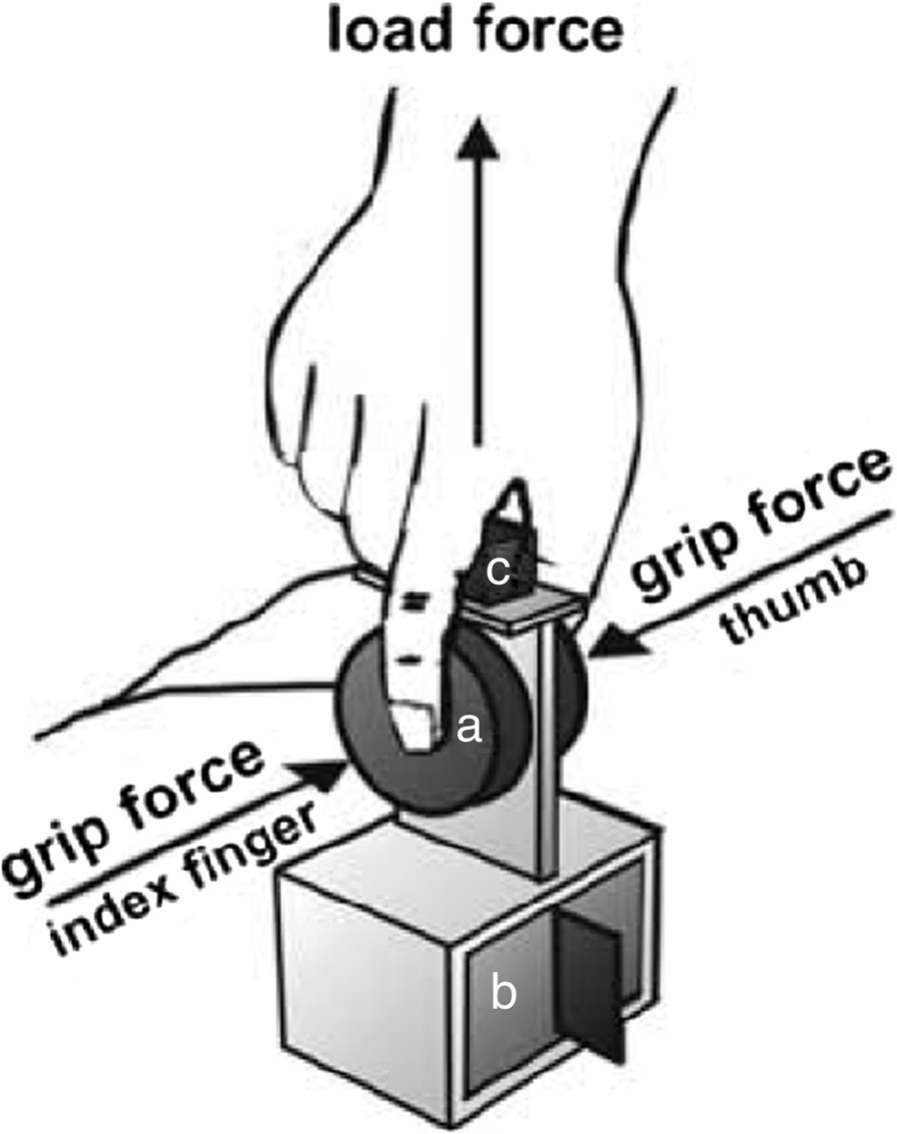
Experimental setup. Legend: Set-up of the Q-Motor grip device (**a**) force transducers for measuring the grip forces of the thumb and/or index finger (*here thumb*), (**b**) the exchangeable weights (250 g or 500 g), and (**c**) the 3D position sensor for measuring involuntary movements

Quantitative Grip Force Assessment (QGFA) and Quantitative Involuntary Movement Assessment (QIMA) was performed as previously described in detail [8, 9]. In short, all subjects were seated in an upright position on a chair and asked to grasp and lift a grip instrument with a force-torque sensor (Nano-40, ATI, Apex, NC), which measured the grip (normal) and load (tangential) forces of the thumb (Fig. 1). The instruments’ weight could be modified to 250 g (light) and 500 g (heavy). An electromagnetic position-angle sensor (Fastrack, Polhemus, VT) continuously measured the instruments three-dimensional position (x, y, z) and orientation (roll, pitch, yaw). Patients were asked to lift the instrument and hold it adjacent to a marker 10 cm high for 15 s (static holding phase). Patients performed five consecutive recorded trials with each object weight (light and heavy) and with both, their dominant and non-dominant hand. Completion of Q-motor tasks required 5 min per hand and weight.

The mean isometric grip force (GF) was calculated as the average during the static holding phase and the five repetitions. Furthermore, the amount of involuntary movement during the static holding phase was assessed by recording changes in position (x, y, z) and orientation (roll, pitch, yaw). To assess the mean amount of involuntary movement in all three dimensions, means of the absolute values of the derivatives of the x, y, and z channels (i.e. velocities occurring in each of the three possible directions in space, expressed in cm/second) were calculated and summed up to the position index (PI). The means of the absolute values of the derivatives of the roll, pitch, and yaw channels (i.e. velocities occurring in each of the two possible angles of rotation in space, expressed in °/second) were added and summed up to the orientation index (OI) [9].

### Statistical analysis

Comparisons between patient (sub-) groups and HC were performed using analysis of covariance (ANCOVA). We included age as covariate in all ANCOVA analyses to adjust for even minor age related effects. All variables were sufficiently normally distributed (unimodal, |skewness| <1) and are reported using means and standard deviations (SDs), except for disease duration for which we report median and interquartile range (IQR). To detect associations among Q-Motor measures (GF, PI, OI) and disease severity as measured by the QMG score, we ran bivariate correlations (univariate analyses) and report the Pearson coefficients.

For all analyses, statistical significance was accepted at the *P* ≤ 0.05 level (2-tailed). Patients with missing values on a specific Q-Motor parameter were case-wise excluded from the specific analysis. The number of patients with missing values ranged in MG patients from 2.5 % for PI of the non-dominant hand and light weight to 7.5 % for PI and OI of the dominant hand and the heavy weight and in HC from 0 % for the PI of the non-dominant hand and light weight to 8.7 % for PI and OI of the non-dominant hand and heavy weight. Statistical tests were performed using SPSS 19 (IBM, Amonk, NY, USA).

### Ethics

The study was approved by the ethics committee of the Charité – Universitätsmedizin Berlin (EA1/281/10). All patients gave written informed consent in accordance with the Declaration of Helsinki in its currently applicable form.

## Results

### Baseline characteristics

Overall, 40 patients with confirmed diagnosis of MG were included. Mean age was 55.7 years, 17 (43.6 %) were female. 34 (85 %) patients were positive for acetylcholine receptor autoantibodies (anti-AChR), the remaining patients were seronegative for anti-AChR and anti-MusK. Seven (17.5 %) patients had clinically OMG (MGFA I, one patient with isolated ptosis, two patients with isolated diplopia and four patients with ptosis and diplopia). Mean disease severity as measured by the QMG score was 7.4 points, median disease duration was 5.5 years. For further details on baseline characteristics stratified by MGFA classification see Table 1.

**Table 1:** Baseline characteristics

Characteristic	MG total	OMG	MGFA IIa	MGFA II + IIIb	Healthy controls
*N*	40	7	18	15	23
Age, y, mean (SD)	55.7 (18.4)	58.3 (19.2)	52.1 (16.0)	58.7 (21.2)	52.0 (16.2)
Female Sex, *n* (%)	17 (43.6)	2 (28.6)	8 (44.4)	7 (46.7)	11 (47.8)
AChR-ab, (%)	34 (85)	5 (71.4)	16 (88.9)	13 (86.7)	n.a
QMG-score, mean (SD)	7.4 (6.3)	2.9 (3.0)	5.7 (5.4)	11.1 (6.5)	n.a.
Disease duration, y, median (IQR)	5.5 (2.25–14.5)	2.0 (2.0–3.0)	6.5 (3.75–30.25)	8.0 (2.0–13.0)	n.a.
Medication					n.a.
ChE-Inhibitors, *n* (%)	31 (77.5)	5 (83.3)	14 (77.8)	12 (80)	n.a.
Steroids, *n* (%)	22 (55.0)	3 (50)	9 (50)	10 (66.7)	n.a.
Immunosuppression, *n* (%)	25 (62.5)	1 (16.7)	12 (66.7)	12 (80)	n.a.

*MG* myasthenia gravis, *HC* healthy controls, *AchR-ab* acetylcholine receptor antibodies, *ChE* cholinesterase, *y* years, *n.a.* not applicable, *IQR* interquartile range

### Differences between MG patients and healthy controls

The position index (PI) and the orientation index (OI) were significantly higher in MG patients compared to HC for both weights in the dominant and non-dominant hand. No differences between MG patients and HC were found for isometric grip force (for details see Table 2).

**Table 2:** Mean values and group comparisons HC vs. MG patients

Parameter	MG	HC	*p*-value	Partial eta square
	Mean (SD)	Mean (SD)		
Isometric grip force 500 g_non-dominant (N)	8.6 (2.9)	8.6 (2.6)	0.93	0.00
Position index 500 g_non-dominant (cm/s)	2.7 (1.2)	1.6 (0.7)	<0.001	0.22
Orientation index 500 g_non-dominant (°/s)	8.6 (4.6)	4.8 (2.1)	0.001	0.19
Isometric grip force 500 g_dominant (N)	8.5 (3.7)	9.3 (3.7)	0.34	0.02
Position index 500 g_dominant (cm/s)	2.7 (1.1)	1.6 (0.6)	<0.001	0.24
Orientation index 500 g_dominant (°/s)	6.9 (2.3)	4.3 (1.6)	<0.001	0.27
Isometric grip force 250 g_non-dominant (N)	6.0 (2.7)	6.7 (3.7)	0.26	0.04
Position index 250 g_non-dominant (cm/s)	1.6 (0.6)	1.0 (0.4)	<0.001	0.24
Orientation index 250 g_non-dominant (°/s)	8.1 (3.3)	4.9 (2.3)	<0.001	0.21
Isometric grip force 250 g_dominant (N)	5.8 (2.3)	6.4 (2.9)	0.29	0.02
Position index 250 g_dominant (cm/s)	1.8 (0.7)	1.1 (0.4)	<0.001	0.27
Orientation index 250 g_dominant (°/s)	7.2 (4.1)	4.3 (2.1)	0.003	0.15

Means and standard deviation (in parentheses) of Q-Motor grip force and involuntary movement assessments. *HC* healthy controls, *MG* myasthenia gravis

### Correlation with QMG

**Fig. 2:**
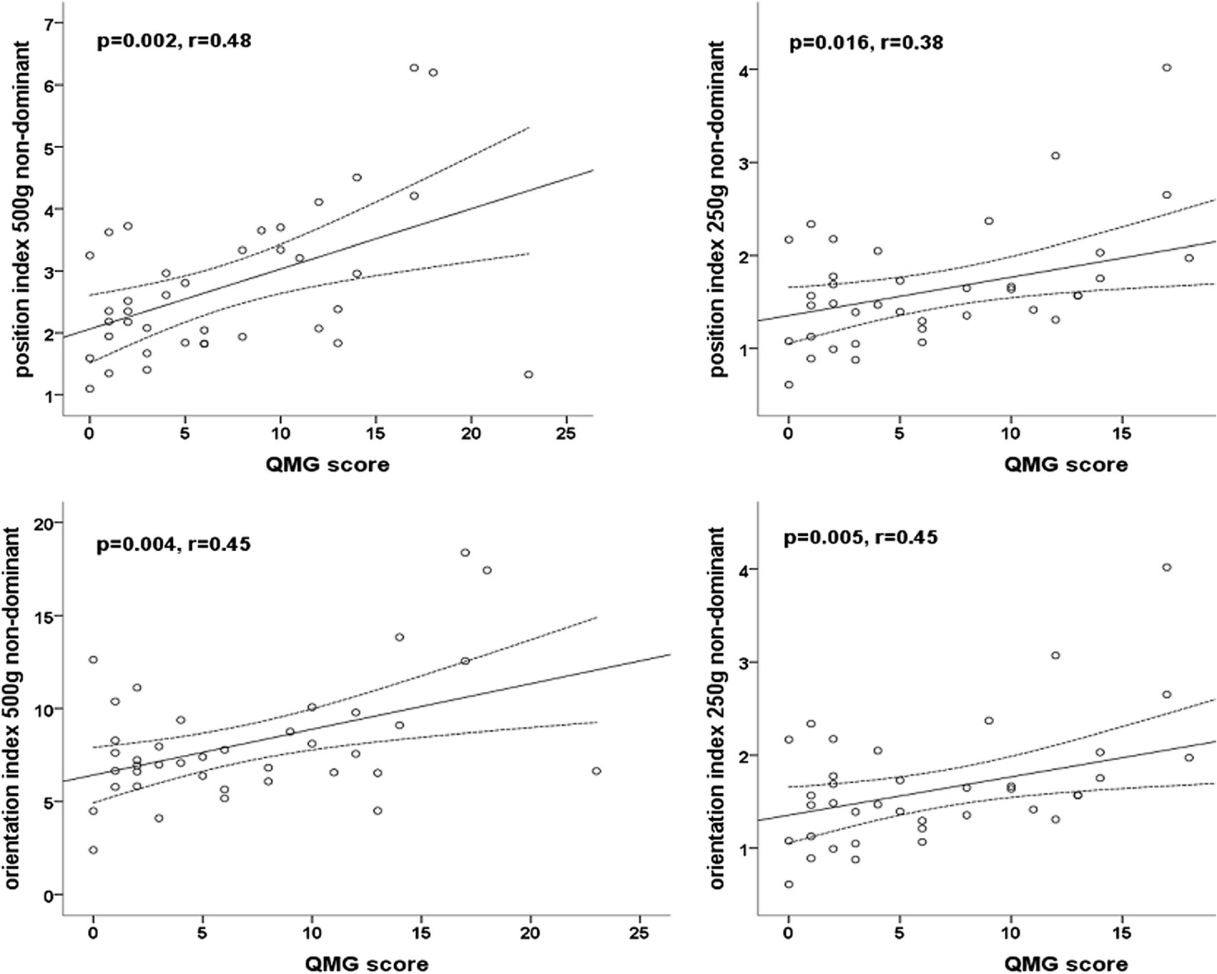
Q-Motor involuntary movement measures correlations with QMG score. Legend: Correlations of position and orientation indices with quantitative myasthenia gravis score for the light and heavy weight in the non-dominant hand. QMG = quantitative myasthenia gravis score, *p* = *p*-value, *r* = Pearson correlation coefficient, solid line = fit line, dotted line = 95 % CI

For all parameters that were significantly higher in MG patients, we assessed the correlation with the QMG-score. PI and OI correlated significantly with QMG for both weights in the non-dominant hand (for details see Fig. 2). In the dominant hand, only OI for the light weight correlated significantly with QMG (Pearson correlation coefficient *r* = 0.42, *p*-value = 0.009, data not shown).

### MGFA subgroup analysis

Patients with purely ocular symptoms (MGFA I) showed significantly higher values for the PI and OI in the dominant and non-dominant hand for both weights compared to HC (Table 3). Of the seven patients with OMG, 5 (71.4 %) had acetylcholine receptor autoantibodies and 2 (28.6 %) were seronegative. Median disease duration in OMG patients was 2.0 years, one patient had a disease duration of 1 year, three patients had a disease duration of 2 years, two patients had a disease duration of 3 years, and one patient had a disease duration of 11 years. Additionally, we assessed differences in grip force tasks in the subgroups of patients with generalized MG. There were no significant differences between patients with muscle weakness predominantly affecting limb and/ or axial muscles (MGFA IIa) and patients with muscle disease predominantly affecting oropharyngeal and/or respiratory muscles (MGFA IIb + IIIb) (Table 3).

**Table 3:** Subgroup analysis of statistically significant Q-Motor involuntary movement measures

Parameter	HC vs. OMG			MGFA IIa vs. IIb		
	OMG	HC	*p*-value (h_p_^2^)	MGFA IIa	MGFA II + IIIb	*p*-value (h_p_^2^)
	Mean (SD)	Mean (SD)		Mean (SD)	Mean (SD)	
Position index 500 g_non-dominant (cm/s)	2.5 (0.7)	1.6 (0.7)	0.004 (0.30)	2.9 (1.4)	2.8 (1.3)	0.47 (0.02)
Orientation index 500 g_non-dominant (°/s)	8.6 (2.3)	4.7 (2.1)	<0.001 (0.45)	9.1 (4.7)	8.7 (5.8)	0.26 (0.06)
Position index 500 g_dominant (cm/s)	3.0 (1.7)	1.6 (0.6)	0.003 (0.33)	2.9 (1.1)	2.5 (0.8)	0.39 (0.03)
Orientation index 500 g_dominant (°/s)	7.7 (2.5)	4.2 (1.6)	<0.001 (0.44)	7.0 (2.3)	6.8 (2.2)	0.94 (0.00)
Position index 250 g_non-dominant (cm/s)	1.6 (0.5)	1.0 (0.4)	0.001 (0.39)	1.7 (0.8)	1.6 (0.6)	0.60 (0.01)
Orientation index 250 g_non-dominant (°/s)	7.9 (2.7)	4.9 (2.3)	0.007 (0.27)	8.3 (3.7)	8.1 (3.6)	0.36 (0.04)
Position index 250 g_dominant (cm/s)	2.1 (0.7)	1.1 (0.4)	0.001 (0.40)	1.8 (0.7)	1.9 (0.8)	0.83 (0.00)
Orientation index 250 g_dominant (°/s)	7.5 (2.2)	4.3 (2.1)	0.010 (0.25)	6.4 (2.4)	8.7 (6.0)	0.50 (0.02)

*OMG* ocular MG, *HC* healthy controls, *MGFA* myasthenia gravis Foundation of America classification, *h*_*p*_^*2*^ partial eta squared

## Discussion

In this cross-sectional pilot study we investigated 40 MG patients using quantitative motor (Q-Motor) grip force assessment (QGFA) and involuntary movement assessment (QIMA) and compared their results to 23 HC of comparable age and sex. Our aim was to objectively quantify muscular weakness in MG patients. We demonstrated that PI and OI are significantly higher in MG patients compared to HC. Furthermore, subgroup analysis revealed that MG patients with purely ocular symptoms also showed significantly higher values for PI and OI compared to HC.

Assessment of muscle weakness is crucial for clinical monitoring of MG patients, evaluation of treatment success and as outcome parameter in clinical trials. However, so far, no objective measure for MG disease severity has been established. Recommendations for clinical research standards published by the Task Force of the Medical Scientific Advisory Board of the Myasthenia Gravis Foundation of America comprise the use of the MGFA classification to identify subgroups of patients with MG who share distinct clinical features or severity of disease [3]. However, it is not recommended as outcome parameter in clinical trials. Further limitations of the MGFA classification are the subjective assessment and therefore inherent imprecision as well as the lack of quantification [3]. The QMG score is a more objective tool to assess disease severity reliably, has proven a good interrater reliability [4] and is recommended to be used in all prospective interventional studies for MG [3]. Yet, quantification of the QMG score is limited (graduation of 0–3 points, with three being the most severe) [10] and should not be used to compare severity between patients. Currently, the MGFA Postintervention Status is recommended to be used after starting MG treatment [3]. However, like the MGFA classification, the MGFA Postintervention Status is a subjective classification with an inherent imprecision and does not allow for comparison with the “preintervention” status. QGFA and QIMA allow an objective assessment of muscular weakness in patients with MG.

The herein used Q-Motor quantitative grip force assessments (QGFA) and quantitative involuntary movement assessment (QIMA) have been shown to detect involuntary movements in subjects with premanifest Huntington’s disease, to correlate with disease severity in patients with manifest Huntington’s disease and to increase with disease progression [9, 11–13]. Recently, these measures have successfully been applied in a randomized controlled clinical trial for the treatment of chorea in huntington’s disease and exhibited a higher sensitivity than clinical rating scales and, notably, no placebo effects [14].

In our MG patients, PI and OI were significantly higher for the lighter and heavier weight in the dominant and non-dominant hand compared to HC. Interestingly, isometric GF as a measure for overt muscular weakness did not differ significantly in MG patients compared to HC. We hypothesize that PI and OI as measures for involuntary movements in space are more sensitive parameters for myasthenic muscular weakness and resulting deficits in motor task performance. While muscle strength can still be enough to hold the weight, it might not be enough to hold it steadily. Just like a weightlifter starts shaking before dropping the weight. Therefore, PI and OI might be suitable to detect subclinical affection of myasthenic weakness in otherwise clinically unaffected muscles.

Accordingly, PI and OI were significantly higher for both weights and both hands in patients with clinically ocular myasthenia gravis (OMG). Extraocular symptoms are the most common presenting sign of MG. More than 50–60 % of patients with inital OMG develop generalized myasthenia gravis (GMG) over the course of their illness [15, 16]. To date, no clinical or laboratory parameters are available that allow for an identification of OMG patients at high risk for generalization of MG. OMG patients with normal SFEMG are unlikely to generalize, however, abnormal SFEMG is not predictive of subsequent development of GMG [17]. A predictive parameter could justify therapy escalation in OMG patients, e.g. with glucocorticoids that seem to decrease the risk of generalization of MG [18]. QGFA and QIMA have been shown to be able to detect motor signs in subjects with premanifest Huntington’s disease [13, 19]. We therefore plan to assess the predictive properties of Q-Motor QGFA and QIMA for generalization of OMG in a prospective longitudinal clinical study.

No differences in PI and OI value were seen between patients with predominantly affected limb and/or axial muscles and patients with predominantly affected oropharyngeal and/ or respiratory muscles. The explanation for this finding might be twofold: Firstly, to assess differences between subgroups of MG-patients, all patients were categorized into either MGFA IIa or MGFA IIb + IIIb. Categorization based on the clinical judgement of the study physician concerning the pattern of muscle weakness. Thereby, the predominantly but not exclusively affected muscle groups decided upon MGFA classification. The fluctuating extent and variable predominance of the muscle groups involved, makes it extremely difficult to classify MG patients and the inherent imprecision of the MGFA classification is widely accepted [3]. Secondly, Q-Motor might be able to detect subclinical affection of limb muscle weakness in patients with predominantly affected oropharyngeal and/or respiratory muscles the way it seems to do in patients with purely ocular symptoms.

The study has several limitations. The small sample size, especially in the subgroup analysis, must be acknowledged. However, despite the limited number of patients with OMG, alterations in OI and PI reached statistical significance. It has to be mentioned that OMG patients had a shorter disease duration compared to patients with GMG (Table 1). Therefore, we can not exclude that worse motor task performance in OMG patients compared to HC was due to a beginning, subclinical generalization of myasthenic symptoms. However, six out of seven patients had a disease duration of 2 years or longer. Risk for generalization of MG is highest within the first 3 years, approximately 80 % of patients generalize within the first 2 years after symptom onset [20, 21]. Hence, it is unlikely that the shorter disease duration entirely explains the differences seen between OMG patients and HC. The fact, that PI and OI values in OMG patients did not correlate with disease duration seems to support this assumption (data not shown). An extended follow-up testing of OMG patients will be performed in subsequent studies.

No differences were seen for PI and OI between OMG and GMG-patients. There might be two possible explanations for this finding. The missing differences between OMG and GMG patients could be due to the hypothesized subclinical affection of limb muscles in OMG patients. Another explanation might be the patient selection. Though inclusion criteria encompassed all MG-patients independent of disease severity excluding only myasthenic crisis, the GMG-patients enrolled were only mildly affected (all patients but one patient had MGFA classification IIa/b). Future studies on Q-Motor Assessment will have to include more severely affected patients to show plausible differences between OMG and GMG-patients.

Patients were recruited for Q-Motor assessment at our outpatient clinic and were allowed to take their MG-specific medication without a specific time interval prior to testing. Cholinesterase inhibitors are prone to influence motor task performances although most likely by attenuating muscular weakness.

Comparability and, thus, correlation of QMG score and Q-Motor measures might be limited. Firstly, QGFA and QIMA assess only one body region whereas QMG score assesses 13 items involving different muscle groups. Future studies on Q-Motor Assessment should compare correlations of total QMG score and its subitems (i.e. arm outstretched, hand grip). Secondly, QGFA and QIMA mainly assess more distal muscle groups whereas muscle strength of limb muscles addressed by the QMG score are more proximally located. This might partially explain the inconsistent correlations of performance in QGFA/QIMA and QMG score.

A comparison of Q-motor assessment with other electrophysiological tests such as repetitive nerve stimulation (RNS) and single-fiber electromyography (SFEMG) was not part of this pilot study but will be subject in future investigations. Furthermore, we plan to correlate Q-motor assessment not only with muscle fatigability but also with fatigue as a subjective sensation of exhaustion and frequent phenomenon in MG [22, 23].

## Conclusions

Q-Motor QGFA and QIMA assessments might be useful tools to quantitatively assess muscular weakness in MG. Q-Motor assessments are safe, noninvasive, easily applicable methods that can be used in outpatient settings. If QGFA/QIMA prove their validity in a larger cohort, they might be valid tools to use as endpoints increasing the sensitivity and power of future clinical trials in MG. Furthermore, QGFA/QIMA might help to identify OMG patients at high risk for generalization of MG.
